# A robust method for automatic identification of femoral landmarks, axes, planes and bone coordinate systems using surface models

**DOI:** 10.1038/s41598-020-77479-z

**Published:** 2020-11-30

**Authors:** Maximilian C. M. Fischer, Sonja A. G. A. Grothues, Juliana Habor, Matías de la Fuente, Klaus Radermacher

**Affiliations:** Helmholtz-Institute for Biomedical Engineering, RWTH Aachen University, Pauwelsstr. 20, 52074 Aachen, Germany

**Keywords:** Bone, Bone quality and biomechanics, Software, Image processing

## Abstract

The identification of femoral landmarks is a common procedure in multiple academic fields. Femoral bone coordinate systems are used particularly in orthopedics and biomechanics, and are defined by landmarks, axes and planes. A fully automatic detection overcomes the drawbacks of a labor-intensive manual identification. In this paper, a new automatic atlas- and a priori knowledge-based approach that processes femoral surface models, called the A&A method, was evaluated. The A&A method is divided in two stages. Firstly, a single atlas-based registration maps landmarks and areas from a template surface to the subject. In the second stage, landmarks, axes and planes that are used to construct several femoral bone coordinate systems are refined using a priori knowledge. Three common femoral coordinate systems are defined by the landmarks detected. The A&A method proved to be very robust against a variation of the spatial alignment of the surface models. The results of the A&A method and a manual identification were compared. No significant rotational differences existed for the bone coordinate system recommended by the International Society of Biomechanics. Minor significant differences of maximally 0.5° were observed for the two other coordinate systems. This might be clinically irrelevant, depending on the context of use and should, therefore, be evaluated by the potential user regarding the specific application. The entire source code of the A&A method and the data used in the study is open source and can be accessed via https://github.com/RWTHmediTEC/FemoralCoordinateSystem.

## Introduction

The detection of osseous landmarks of the femur and additional parameters of a higher order, such as axes and planes, is a common process in orthopedics^1–3^, biomechanics^4–6^, morphometrics^7^, anthropometry^8^, epidemiology^9,10^, anthropology^11^ or forensics^12,13^. The manual identification of landmarks necessitates anatomical training of the examiners, is subject to intra- and interobserver variability^14^, is costly in terms of time^15,16^, and unsuitable for the analysis of large datasets. Hence, an automatic identification of landmarks is preferable. However, automatic methods for landmark detection should provide reproducible results, be robust against the spatial alignment of the femur and handle the large interindividual variability^17^ of the femoral morphology.

For the sake of clarity, this paper deals with the fully automatic identification of femoral landmarks and parameters based on a three-dimensional (3D) surface model. Manual and semi-automatic methods are not considered. A possible application scenario is the investigation of large datasets obtained from different sources. In such a case, reasons related to data protection might prohibit the transfer and use of the original volume data. The source of the surface models can be any accurate medical imaging modality and reconstruction technique. However, a robust identification method has to consider that the anatomical planes of the patient can differ highly from the coordinate system of the medical imaging system and that the anatomical orientation of the coordinate system of the medical imaging system can vary.

### Related work

Multiple methods have been proposed in literature for the automatic detection of osseous landmarks and parameters of the femur. They can be categorized into statistical^18–24^ and geometrical approaches^2,17,25–29^ and a combination of both approaches is also possible. The advantage of the statistical approaches is the application of the statistical models in other related areas, such as segmentation^30^ or 3D reconstruction from sparse imaging data^31^. The main drawback of statistical approaches is that they require a sufficient amount of labeled training datasets to build a statistical model. Different geometrical approaches based on a priori knowledge^2,25–29^ and/or labeled atlases^17^ can be used to avoid manual labeling of training datasets. A distinction must be made between a single or multi-atlas approach for the atlas-based methods. A single atlas is based on one labeled generic template model or a single subject, whereas a multi-atlas is generated from multiple datasets and has to be classified as a statistical approach^21,23^. To the best of our knowledge, Kim et al. introduced the first fully automatic geometrical approach to measure femoral neck anteversion in 2000^25^. The method uses geometrical properties, least squares fitting and iterative minimization methods to detect the head center, neck axis, shaft axis, posterior condylar axis and table top plane (TTP) of the femur^25^. The method was validated against manual physical dry bone measurements and compared to other manual methods measuring femoral anteversion in the raw CT volume data. However, a validation against manual measurements on the point cloud data was not performed. Cerveri et al. published a similar geometrical method to identify parameters of the proximal femur for total hip arthroplasty^27^. The heuristic method uses geometrical properties, least squares fitting and evolutionary optimization to identify the femoral head center (FHC), neck axis and shaft axis^27^. The method was validated against manual measurements of three observers. Subburaj et al. presented a geometrical method to assess deformities of the femur for surgical planning^2^. The method localizes possible landmarks based on the 3D curvature of the femoral surface. Subsequently, specific landmarks are identified and labeled in an iterative process based on a spatial adjacency network graph formed between the localized landmark points^15^. The landmarks are used to define the distal condylar and epicondylar axis. In addition, least squares fitting and skeleton extraction are used to detect the FHC, mechanical axis, neck axis and shaft axis for the quantification of femoral torsion or bowing^2^. However, Subburaj and colleagues' landmark detection was only validated against manual measurements at the distal femur for three subjects and the initialization of the spatial adjacency network graph remains unclear^15^. Gharenazifam et al. proposed a geometrical approach to extract a compact 3D skeleton of the femur based on clinical parameters serving as a tool for orthopedic research and clinical issues^28^. The method uses geometrical properties, principal component analysis, least squares fitting and two-dimensional curvature analysis to create the skeleton of the femur consisting of the head, neck, shaft, lesser and greater trochanter, and the distal part, including the posterior condylar and shaft axis. However, a validation of the method against manual measurements was missing in the study. Kai et al. presented a method specifically for the construction of a femoral bone coordinate system using the principal axes of inertia, contour tracking and spherical least squares fitting to the femoral head and posterior condyles^29^. The centers of the spheres are used to construct the coordinate system. The results were compared to a manual identification of the femoral coordinate system recommended by the International Society of Biomechanics that will be referred to as the Wu2002 coordinate system hereinafter^32^. However, the Wu2002 coordinate system is defined by the medial and lateral epicondyle and not by the centers of the posterior condyles, which has led to significant differences between the automatic and manual method. Furthermore, the method was only applied to five femora. Hence, the robustness of the method is difficult to assess. Phan et al. introduced a geometrical approach based on a single atlas to predict landmarks and bony features of the proximal and distal femur for clinical and biomechanical applications^17^. The atlas contains local regions and landmarks that were manually identified on a template model of the proximal and distal femur. The proximal and distal femora are separately processed using the same workflow. The latter starts with a two-stage registration of the template to the subject's model in the Cartesian and spherical parameter space using a scaling iterative closest point and spherical thin-plate spline algorithm. Subsequently, the local regions of the template are mapped to the subject and the latter is subdivided into the local regions. The registration of each local region of the template and subject is repeated using the scaling iterative closest point algorithm. Finally, the regions and landmarks are mapped to the subject to determine the head center, neck axis and shaft axis. The method was validated against manual measurements for 35 subjects. However, the manual measurement was only performed once per subject by one observer.

In summary, multiple geometrical approaches have been presented in literature. However, some of the studies investigated only the proximal and/or distal femur, other studies identified only one single parameter or a limited number of landmarks and in most of the studies, the validation against manual expert measurements as ground truth was insufficient. The differences between femoral coordinate systems constructed from automatic and manual identified landmarks have been especially neglected, although these bone coordinate systems are commonly used in orthopedics and biomechanics.

Therefore, in this paper, we introduce a fully automatic geometrical approach, hereafter referred to as the A&A method, for the identification of landmarks on a surface model of the femur. The method identifies the landmarks and parameters to construct three established femoral coordinate systems, including the one recommended by the International Society of Biomechanics^32^. We hypothesized that the A&A method robustly identifies the femoral landmarks and parameters independently from the initial spatial alignment of the femoral surface model and without significant differences to a manual identification.

## Materials and methods

A list of all abbreviations used in this paper and a figure of the detected landmarks, axes and planes that are used to construct the femoral bone coordinate systems can be found in the Supplementary Table S1.

### Subject data

The CT data of twenty cadaveric subjects from the open source virtual skeleton database^33^ hosted at the SICAS Medical Image Repository (www.smir.ch) were used in this study (Table 1). Subjects with obvious bone fractures or metal artifacts in the region of the femora were not included in the study. Ten left and ten right femora were selected in alternating order from the twenty cadaveric subjects. Additional information for each subject can be found in the Supplementary Table S2. The surface of each femur was semi-automatically reconstructed using a workflow described previously^34,47^.

**Table 1:** Gender and age of the cadaveric subjects.

Gender	Number of subjects	Age		
		Median	IQR (Q1 to Q3)	Range (min. to max.)
Male	13	58	35 (38 to 73)	59 (25 to 84)
Female	7	41	14 (38 to 52)	31 (30 to 61)

### Femoral landmark identification

#### A&A method

**Figure 1:**
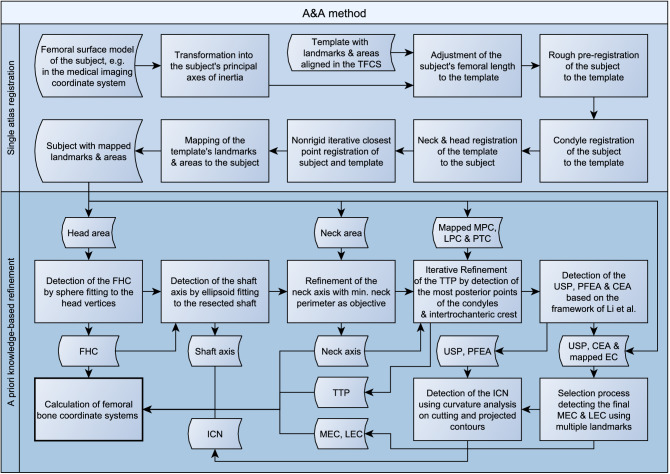
Flowchart of the A&A method. The A&A method starts with a single atlas-based registration (top) followed by a refinement of the landmarks, axes and planes that are used to construct the femoral bone coordinate systems (bottom).

The A&A method is divided into a single atlas-based approach in a first step, followed by a refinement of the landmarks, axes and planes based on a priori knowledge in a second step. The surface model and the side of the femur have to be passed to the program. A general overview of the A&A method is depicted as a flowchart in Fig. 1 and a detailed description is reported below. The MATLAB source code is publicly archived^42^ and can be consulted for every small detail about the method. Future updates can be found at https://github.com/RWTHmediTEC/FemoralCoordinateSystem.

The single atlas-based approach starts with a nonrigid registration of a femoral template surface (template). The femoral surface of the subject (subject) is translated to its center of mass^35^ and rotated to its principal axes of inertia, the eigenvectors of the tensor of inertia sorted by the eigenvalues in descending order^29^. Due to the morphology of the femur, this leads in most cases to the necessary orientation of the temporary femoral coordinate system (TFCS), as specified in Table 2. However, the transformation to the principal axes of inertia does not guarantee the orientation required for every femur due to the large interindividual variability of the femoral morphology.

**Table 2:** Orientation of the TFCS required after the rotation of the femur to its principal axes of inertia.

Axis	x	y	z
Negative direction	Distal	Medial	Posterior
Positive direction	Proximal	Lateral	Anterior

**Figure 2:**
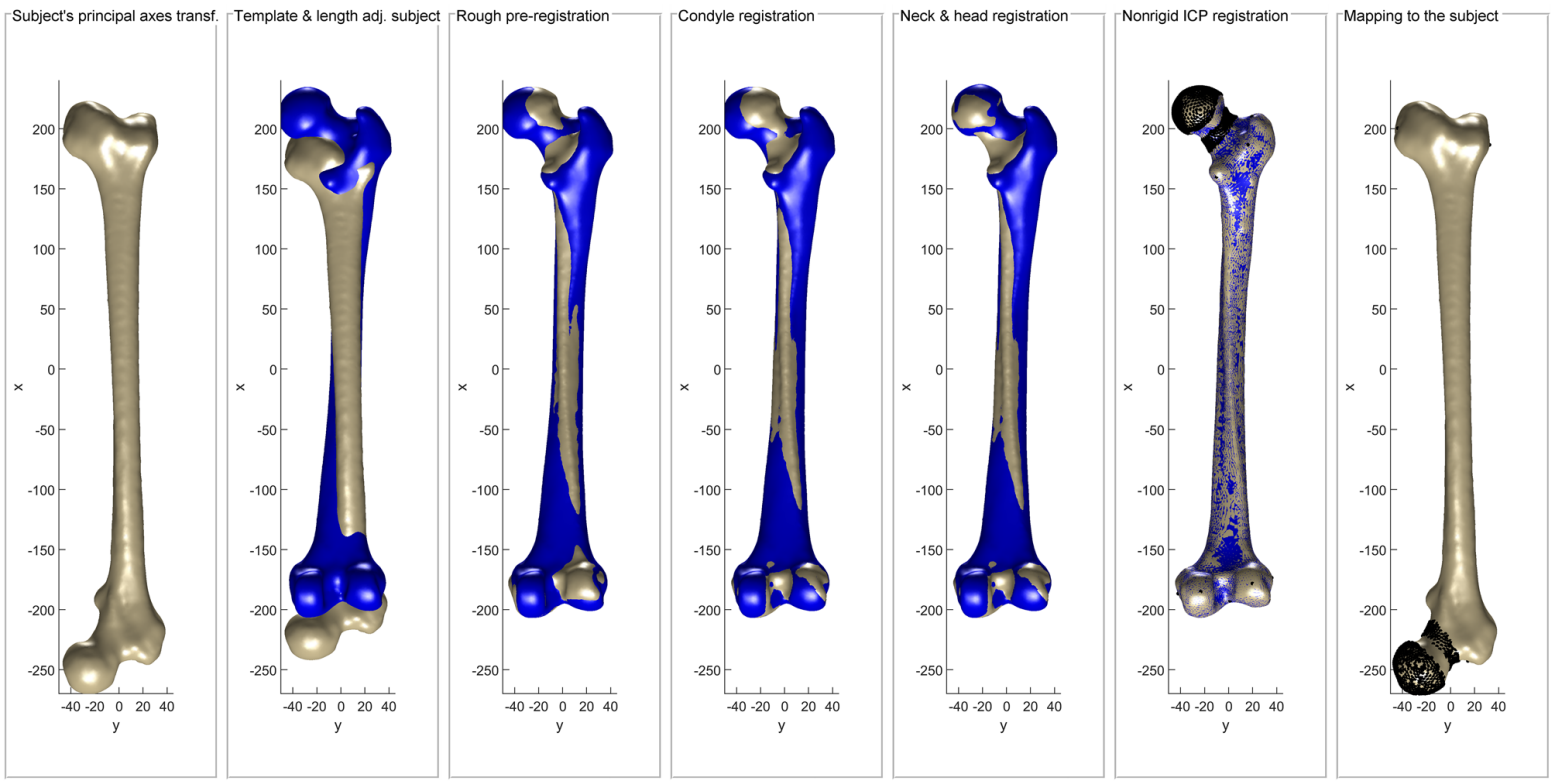
Registration process of the subject (grey) and template (blue), including the final mapping of landmarks and areas (black) from the template to the subject.

The template is already aligned to the TFCS and the long axis (x-axis) of the subject is scaled to the length of the template. A rough pre-registration now follows. The subject is rotated along the x-axis with a step size of 10°. This is repeated for a 180° rotation around the y-axis in case the subject is positioned upside down in the TFCS. The sum of the distances between the nearest neighbors of the template and subject is calculated for each step and the rotation with the smallest sum is chosen as a rough pre-registration. The rotation along the x-axis is repeated with a step size of 5° for the distal part of the subject's surface model to align the femoral condyles of the template and subject. Subsequently, the femoral version (also known as the torsion), neck-shaft angle (also known as the CCD angle) and neck length of the template are varied using linear blend skinning deformations^36^ to align the proximal part of the template and subject. Finally, the template is registered to the subject using a nonrigid iterative closest point algorithm^37,^ ^44^ and landmarks and areas of the template, such as the head or neck, are mapped to the subject (Fig. 2).

**Figure 3:**
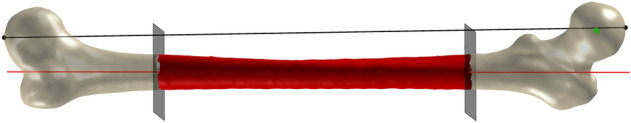
Detection of the shaft axis (red). The vector connecting the points with the maximum distance (black) defines the normal of the two cutting planes (grey) for the resection of the proximal and distal part. The FHC (green) is required to determine the direction of the vector.

The femoral landmarks, axes and planes of the subject can now be determined and refined. Some landmarks, such as the most superior point of the greater trochanter (SGT) or the lesser trochanter (LT) are simply mapped from the template without further refinement. The FHC is calculated by least-squares fitting of a sphere to the mapped vertices of the head. The shaft axis is determined by cropping the femur to the half of its length resecting the distal and proximal part. After least squares fitting of an ellipsoid to the remaining shaft, the major axis of the ellipsoid defines the shaft axis (Fig. 3).

**Figure 4:**
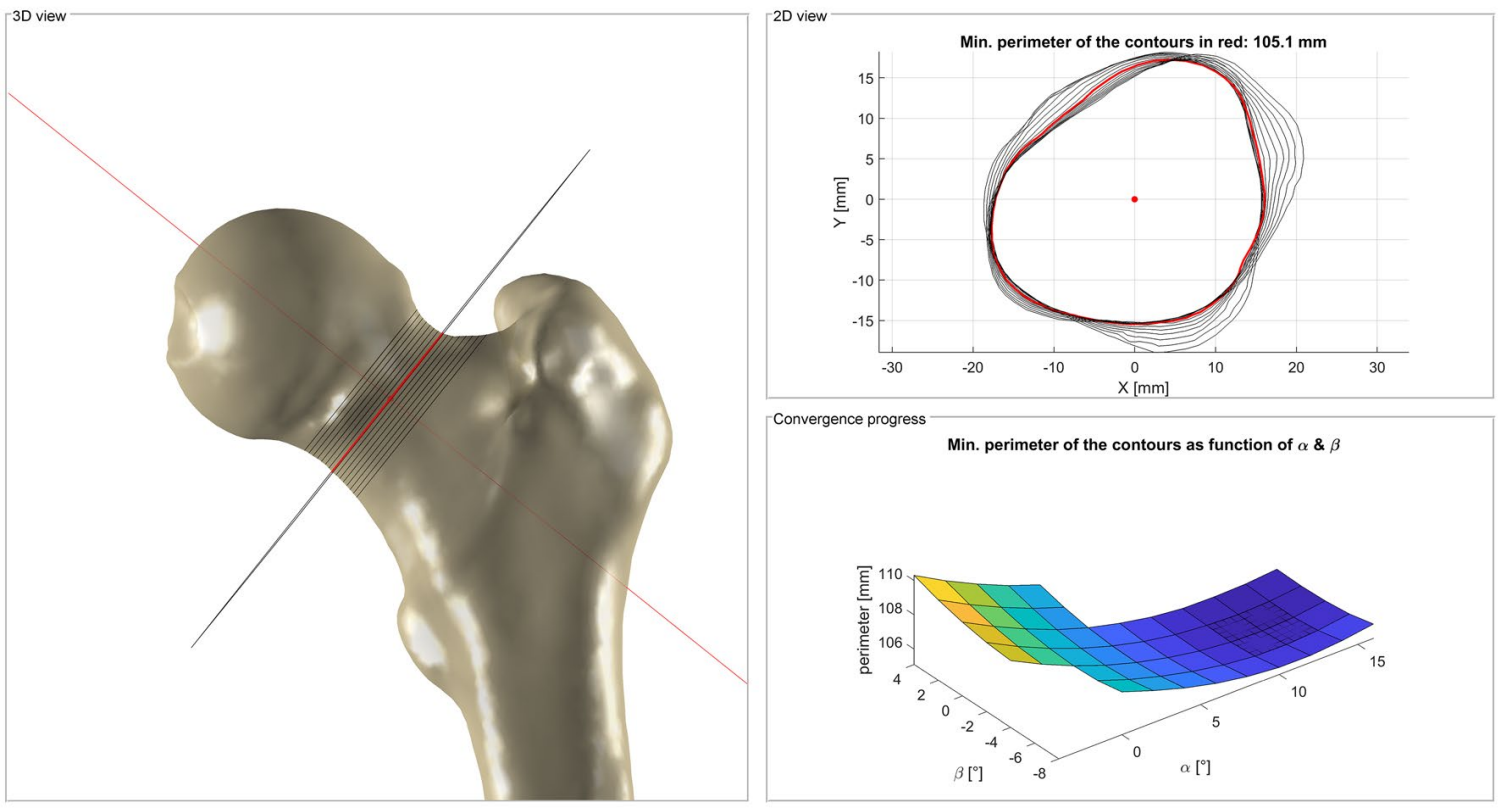
Iterative procedure for the refinement of the neck axis. The orientation of cutting planes through the neck is changed by the angles α and β. The procedure is repeated until the perimeter of one of the cutting contours converges to a minimum^43^.

An initial neck axis is determined by least squares fitting of an ellipse to the mapped area of the neck. The normal of the ellipse serves as a starting point for a refinement of the neck axis by iteratively changing the orientation of cutting planes through the neck. The procedure is repeated until the perimeter of the cutting contours converges to a minimum. The normal of the cutting contour with the smallest perimeter defines the neck axis (Fig. 4)^43^.

**Figure 5:**
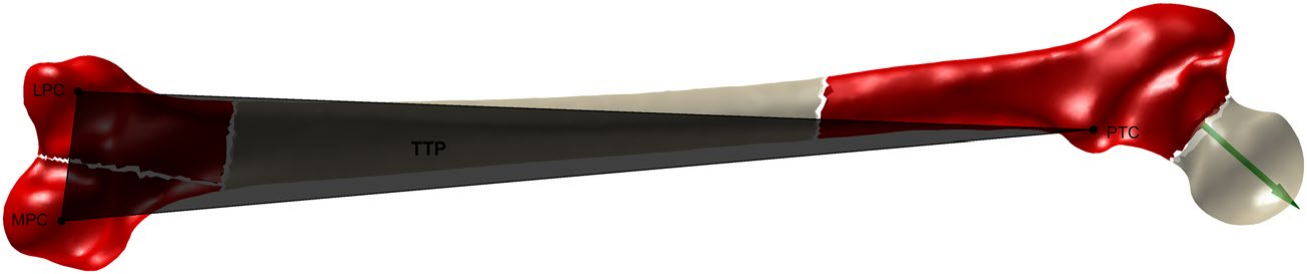
Three parts of the femur (red) are used to iteratively detect the MPC, LPC and PTC to define the TTP (black). The neck axis (green) defines the neck cut for the resection of the head to avoid misdetections of the PTC in case of a retroversion of the neck.

The TTP is formed by three points: the most posterior point of the medial and lateral femoral condyle (MPC, LPC) and the most posterior point of the trochanteric crest (PTC)^38^. Three separate parts of the femur are used for the detection: the medial and lateral condyle and the proximal part without the head resected at the neck. The resection is required to avoid misdetections of the PTC in case of a retroversion of the neck. The most posterior point of each part now defines the temporary MPC, LPC and PTC that define the temporary TTP. All parts are rotated into the coordinate system defined by the temporary TTP and the detection of the landmarks is repeated until the rotation matrix converges to the unit matrix. Hence, the final MPC, LPC, PTC and TTP are identified (Fig. 5).

**Figure 6:**
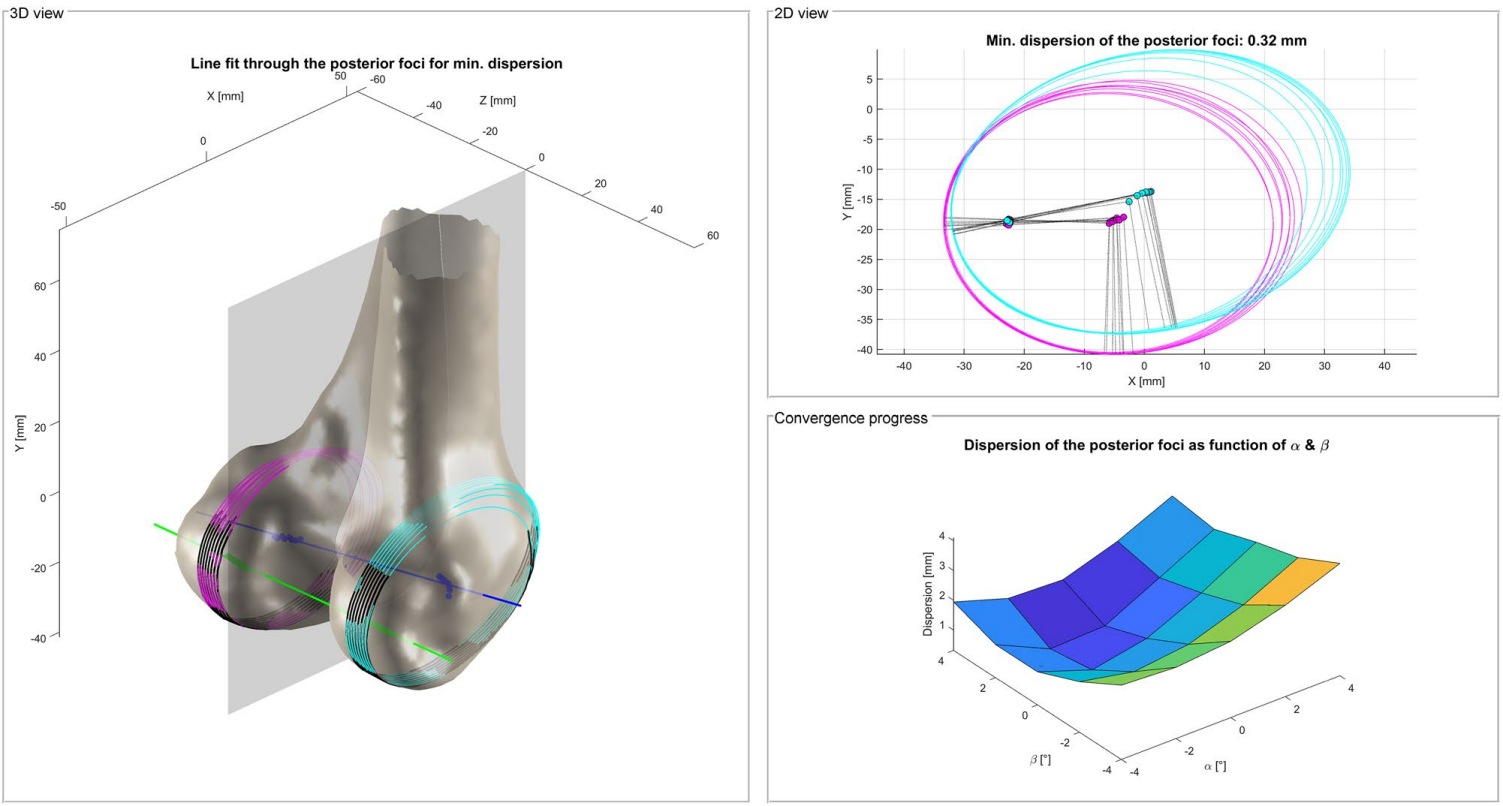
Iterative framework of Li et al.^39^ for the detection of the USP (grey) based on the PFEA (green). The orientation of the sagittal cutting planes through the condyles (medial: magenta; lateral: cyan) is changed by the angles α and β. Ellipses are fitted to the articulating part of the cutting contours until the dispersion of the posterior foci of the ellipses converges to a minimum. The PFEA (green) is fitted to the posterior foci and the CEA (blue) to the centers of the final ellipses^45^.

The medial and lateral epicondyle (MEC, LEC) are detected by a specially designed selection process using multiple landmarks. First of all, the unified sagittal plane (USP) of the condyles is calculated based on an automated framework proposed by Li et al. using only the distal part of the femur^39^. The USP is based on the posterior focal elliptic axis (PFEA) that is fitted to the posterior foci of ellipses that are fitted to the articulating part of the sagittal cutting contours of the femoral condyles. This process is repeated until the dispersion of the posterior foci converges to a minimum (Fig. 6)^45^.

The intersection points of the axis fitted through the centers of the final ellipses (CEA) with the distal part of the femur (EC_CEA_) are two landmarks used in the selection process. The most medial and lateral points in the USP coordinate system (EC_max_) and the mapped epicondyles from the template femur (EC_map_) are additional landmarks. Hence, three landmarks are used for each epicondyle. The EC_max_ are usually taken as the final MEC and LEC. However, this can lead to misdetections in the case of large osteophytes at the medial or lateral rim of the articulating surface. The distance between the EC_CEA_ and EC_map_ and between EC_CEA_ and EC_max_ are compared for each side separately. If the EC_CEA_-EC_map_ distance is smaller, two spheres with the EC_CEA_ and EC_map_ as the center and the EC_CEA_-EC_map_ distance as the radius are clipped from the distal femur and the most medial or lateral point of the clipped part is taken as the final MEC or LEC.

The intercondylar notch (ICN) is identified using a technique adapted from the USP calculation. The intersection points of the PFEA with the condyles divide the distal femur into three sectors: the medial and lateral condyle and the intercondylar sector. The sagittal cutting contours of the intercondylar sector are analyzed in a way similar to the USP algorithm^39^. Based on the curvature, an extreme point is detected in each cutting contour that defines the boundary between the trochlear surface and the intercondylar fossa. The median of the boundary points defines the temporary ICN in the posterioanterior direction. The silhouette of the intercondylar sector in the frontal plane is calculated and the local maximum of the distal outline of the silhouette defines the distoproximal and mediolateral component of the temporary ICN. The nearest neighbor of the boundary points between the trochlear surface and intercondylar fossa to the temporary ICN defines the final ICN^46^.

Three established femoral bone coordinate systems can now be calculated using the landmarks and axes. The coordinate systems investigated in this study are described in Table 3.

**Table 3:** Definition of the orientation of the three femoral bone coordinate systems. The origin of each coordinate system is the FHC.

Name	Wu2002^32^	Bergmann2016^5^	TableTop^38^
1st axis	The epicondylar axis defined by the MEC and LEC	The posterior condylar axis defined by the MPC and LPC	The posterior condylar axis defined by the MPC and LPC
2nd axis	The mechanical axis defined by the midpoint between the epicondyles and the FHC	The straight femur axis defined by the ICN and the point of the neck axis closest to the shaft axis	The normal of the TTP defined by the MPC, LPC and PTC
Distoproximal axis	The mechanical axis	The straight femur axis	The orthogonal of the mediolateral and posteroanterior axis
Mediolateral axis	The orthogonal of the posteroanterior and distoproximal axis	The orthogonal of the posteroanterior and distoproximal axis	The posterior condylar axis
Posteroanterior axis	The orthogonal of the distoproximal and epicondylar axis	The orthogonal of the distoproximal and the posterior condylar axis	The normal of the TTP

#### Manual method

Five experts identified twenty-two landmarks on the femoral surfaces using a graphical user interface implemented in MATLAB. The head was defined by six landmarks and the neck isthmus by four landmarks. Each observer processed all twenty femora and the identification was repeated four times by each observer. Each observer was asked to process a maximum of ten different femora per day. The femora were presented to the observer in the CT coordinate system. The shaft axis was not manually identified but calculated as described in the chapter "A&A method".

### Evaluation

Intraclass correlation coefficients were calculated for the landmarks selected manually in the CT coordinate system to examine the interobserver reliability (two-way random effects, single measures, absolute agreement) and intraobserver reliability (two-way mixed effects, single measures, absolute agreement) of the manual method. More than ten subjects had to be processed by each of the five observers to ensure a desired 95% confidence interval (⍺ = 0.05) with a width of 0.2 considering an estimated intraclass correlation coefficient of 0.9^40^.

The robustness of the A&A method against the initial alignment of the femur in space was evaluated by applying a random transformation (rotation and translation) to the femur in the CT coordinate system and calculating the Wu2002 coordinate system subsequently. The rotation could range from -360° to 360° around each axis and the translation from -1000 mm to 1000 mm along each axis. The procedure was repeated 100 times for each subject.

The manual landmarks were transformed into the Wu2002 coordinate system calculated by the A&A method for the comparison of the manual method with the automatic method and the division of the differences into the anatomical directions within a standardized coordinate system. The distributions of the manual landmarks were tested for normality using the Lilliefors test. The test was rejected for 39% of the variables and, therefore, nonparametric statistics were used hereafter. The median of all observers and all trials was calculated for each landmark and the nearest point on the surface of the femur to the median point was taken as the reference landmark. The reference landmarks were subtracted from the manual landmarks to investigate the intra- and interobserver errors and from the automatic landmarks to evaluate the positional differences between the manual and A&A method. The rotation matrix transforming the coordinate system of the reference landmarks into the coordinate system of the method investigated was decomposed into Euler angles to determine the angular differences. The construction of the coordinate systems is explained in Table 3. The Euler angles describe the differences for the adduction-abduction (x-axis), internal–external rotation (y-axis) and flexion–extension (z-axis). The Wilcoxon rank-sum test (⍺ = 0.05) was used to identify significant differences in the locations of the medians among the methods.

## Results

Based the on lower boundary values of the 95% confidence interval of the intraclass correlation coefficients, the interobserver reliability (min. 0.933) and the intraobserver reliability (min. 0.962) of the manual method can be considered as excellent.

The landmarks detected did not differ among the 100 randomized transformations that were applied to each subject.

The median manual method's differences (MMD) of the Euclidian distance to the reference landmarks ranged from 0.3 mm for the FHC to 4.4 mm for the MEC. Excluding the PTC, SGT and LT, the median differences of the Euclidian distance between the landmarks determined by the A&A method (AMD) and the reference landmarks ranged from 0.4 mm for the FHC to 4.0 mm for the MEC. The AMD of the Euclidian distance reached an exceptionally high value of 25.6 mm for the PTC. The cause of this large difference is explained in the discussion. The AMD of the Euclidian distance reached 6.7 mm and 6.1 mm for the SGT and LT. However, both landmarks were not refined after the mapping and not used for the calculation of the coordinate systems. More detailed results are presented in Table 4.

**Table 4:** Differences between the landmarks detected by the manual and A&A method and the reference landmarks. Manual method's difference (MMD) (n = 400), A&A method's difference (AMD) (n = 20), p values of the Wilcoxon rank-sum test (⍺ = 0.05).

		MMD [mm]			AMD [mm]			
Landmark	Direction	Median	IQR (Q1 to Q3)	Range (min. to max.)	Median	IQR (Q1 to Q3)	Range (min. to max.)	p value
FHC	PA	0	0.2 (–0.1 to 0.1)	1.8 (–0.6 to 1.2)	0.1	0.2 (–0.0 to 0.2)	0.6 (–0.3 to 0.4)	0.039*
	DP	0	0.3 (–0.1 to 0.2)	2.6 (–0.9 to 1.7)	–0.1	0.3 (–0.3 to 0.0)	0.7 (–0.5 to 0.2)	0.010*
	ML	0	0.4 (–0.2 to 0.2)	2.9 (–1.6 to 1.4)	0.1	0.5 (–0.2 to 0.3)	1.2 (–0.5 to 0.7)	0.311
	ED	0.3	0.4 (0.2 to 0.6)	2.1 (0.0 to 2.2)	0.4	0.3 (0.3 to 0.5)	0.7 (0.2 to 0.8)	0.482
SGT	PA	0	5.5 (–3.7 to 1.8)	42.0 (–23.5 to 18.5)	–4.6	6.1 (–10.0 to –3.9)	24.9 (–26.7 to –1.8)	< 0.001*
	DP	–0.3	1.2 (–1.2 to 0.0)	10.5 (–8.8 to 1.7)	–1.8	2.0 (–2.9 to –0.9)	7.3 (–7.2 to 0.1)	< 0.001*
	ML	0	3.2 (–1.6 to 1.6)	28.3 (–12.4 to 15.9)	–5	3.2 (–5.9 to –2.7)	16.4 (–13.8 to 2.6)	< 0.001*
	ED	3.7	5.8 (1.6 to 7.4)	24.8 (0.0 to 24.8)	6.7	7.0 (5.4 to 12.4)	26.7 (3.4 to 30.2)	< 0.001*
LT	PA	0.1	1.9 (–0.7 to 1.2)	10.6 (–3.6 to 7.0)	–1.2	4.3 (–3.3 to 1.0)	10.9 (–6.6 to 4.3)	0.006*
	DP	0	2.6 (–1.2 to 1.3)	14.0 (–6.0 to 8.0)	–3.9	3.1 (–5.4 to –2.3)	14.8 (–12.1 to 2.6)	< 0.001*
	ML	0.1	1.8 (–0.7 to 1.1)	9.6 (–4.8 to 4.8)	4.8	5.9 (1.3 to 7.3)	14.3 (–1.8 to 12.5)	< 0.001*
	ED	2.4	1.9 (1.6 to 3.5)	9.4 (0.0 to 9.4)	6.1	5.4 (4.9 to 10.3)	11.7 (3.4 to 15.1)	< 0.001*
PTC	PA	0.2	1.6 (–0.3 to 1.3)	12.9 (–4.8 to 8.1)	–2.4	2.4 (–4.1 to –1.7)	5.6 (–5.9 to –0.3)	< 0.001*
	DP	–0.2	4.8 (–2.6 to 2.1)	24.1 (–8.5 to 15.6)	8.8	25.3 (–0.3 to 25.0)	57.6 (–28.6 to 29.0)	0.002*
	ML	0.1	3.6 (–1.6 to 2.0)	22.0 (–9.6 to 12.4)	7.6	7.4 (3.4 to 10.8)	42.7 (–22.5 to 20.2)	< 0.001*
	ED	4	3.4 (2.4 to 5.7)	18.0 (0.0 to 18.0)	25.6	18.9 (11.9 to 30.8)	32.2 (3.4 to 35.7)	< 0.001*
MPC	PA	0	0.3 (–0.0 to 0.3)	2.8 (–0.7 to 2.1)	0	0.3 (–0.2 to 0.0)	0.9 (–0.6 to 0.3)	0.003*
	DP	0	2.3 (–0.6 to 1.7)	19.3 (–8.2 to 11.2)	0.6	1.9 (0.0 to 1.9)	3.3 (–0.6 to 2.7)	0.231
	ML	0	3.1 (–1.2 to 1.9)	16.4 (–10.0 to 6.3)	1.9	4.3 (0.0 to 4.3)	9.9 (–4.1 to 5.9)	0.011*
	ED	2.5	1.5 (1.6 to 3.1)	11.9 (0.0 to 11.9)	2.8	2.1 (2.3 to 4.5)	5.9 (0.0 to 5.9)	0.12
LPC	PA	0.1	0.4 (0.0 to 0.4)	3.0 (–0.3 to 2.7)	0	0.2 (–0.1 to 0.1)	0.5 (–0.3 to 0.3)	0.002*
	DP	0	2.5 (–1.1 to 1.4)	20.9 (–9.4 to 11.5)	0.9	1.7 (0.0 to 1.7)	5.7 (–2.8 to 2.9)	0.077
	ML	0	3.5 (–2.0 to 1.5)	15.9 (–8.7 to 7.2)	1.4	2.5 (0.0 to 2.5)	5.1 (–1.6 to 3.5)	0.008*
	ED	2.4	2.2 (1.7 to 3.9)	13.4 (0.0 to 13.4)	2.1	2.0 (0.9 to 2.8)	4.3 (0.0 to 4.3)	0.072
MEC	PA	0	5.0 (–2.8 to 2.2)	21.9 (–12.2 to 9.7)	0.3	3.7 (–1.8 to 1.8)	10.0 (–4.2 to 5.8)	0.505
	DP	–0.2	5.5 (–3.1 to 2.4)	21.7 (–11.3 to 10.4)	–2	7.9 (–5.7 to 2.2)	11.8 (–8.6 to 3.2)	0.171
	ML	0.2	0.7 (–0.1 to 0.6)	5.4 (–1.5 to 3.9)	–0.2	0.4 (–0.4 to 0.0)	2.5 (–1.0 to 1.5)	0.001*
	ED	4.4	4.1 (2.4 to 6.5)	13.2 (0.0 to 13.2)	4	3.2 (3.3 to 6.5)	8.6 (0.0 to 8.6)	0.647
LEC	PA	0	2.2 (–1.1 to 1.1)	16.3 (–10.8 to 5.5)	0	0.5 (–0.2 to 0.4)	5.8 (–1.7 to 4.1)	0.898
	DP	0	2.9 (–1.2 to 1.7)	19.9 (–9.6 to 10.3)	–0.6	1.6 (–1.6 to 0.0)	2.9 (–2.4 to 0.5)	0.013*
	ML	–0.2	0.5 (–0.5 to 0.0)	5.0 (–4.8 to 0.2)	0	0.1 (0.0 to 0.1)	0.4 (–0.2 to 0.2)	< 0.001*
	ED	2.2	2.0 (1.6 to 3.6)	13.1 (0.0 to 13.1)	1.6	2.0 (0.0 to 2.0)	4.1 (0.0 to 4.1)	< 0.001*
ICN	PA	0	3.1 (–1.1 to 2.0)	18.9 (–7.3 to 11.6)	–1.5	1.5 (–2.3 to –0.9)	5.2 (–5.1 to 0.0)	< 0.001*
	DP	–0.1	2.0 (–1.0 to 1.0)	16.9 (–4.3 to 12.6)	0.6	1.1 (0.4 to 1.5)	3.2 (–0.9 to 2.3)	0.001*
	ML	0.1	1.8 (–0.9 to 1.0)	9.7 (–5.4 to 4.2)	2.6	2.6 (1.2 to 3.8)	6.7 (–2.0 to 4.7)	< 0.001*
	ED	2.4	2.4 (1.4 to 3.8)	14.5 (0.0 to 14.5)	3.3	1.6 (2.7 to 4.3)	6.1 (1.1 to 7.2)	0.020*

A significant difference between the MMD and AMD was present for the adduction–abduction of the neck axis. This caused a significant difference of the adduction-abduction of 0.3° for the Bergmann2016 coordinate system. Additionally, a significant difference of 0.2° was present for the flexion–extension. A significant difference of 0.5° between the MMD and AMD was observed for the flexion–extension for the TableTop coordinate system. The dispersion of the MMD was considerably larger than the dispersion of the AMD, particularly for the neck axis (Table 5).

**Table 5:** Differences between the axes and coordinate systems detected by the manual and A&A method and the reference axes and coordinate systems. Manual method's difference (MMD) (n = 400), A&A method's difference (AMD) (n = 20), p values of the Wilcoxon rank-sum test (⍺ = 0.05).

	Rotation	MMD [°]			AMD [°]			p value
		Median	IQR (Q1 to Q3)	Range (min. to max.)	Median	IQR (Q1 to Q3)	Range (min. to max.)	
Neck Axis	IE	0.1	4.8 (–2.0 to 2.8)	24.3 (–10.4 to 13.8)	–0.4	1.7 (–1.1 to 0.6)	8.0 (–5.5 to 2.6)	0.297
	AA	–0.2	4.7 (–2.5 to 2.3)	25.8 (–14.6 to 11.2)	2	2.3 (1.2 to 3.5)	5.7 (–0.7 to 5.1)	< 0.001*
	FE	0.1	4.4 (–1.7 to 2.7)	25.4 (–10.3 to 15.2)	–0.2	1.9 (–1.1 to 0.8)	5.7 (–3.3 to 2.4)	0.525
Wu2002	IE	–0.2	4.1 (–2.4 to 1.7)	21.3 (–8.9 to 12.4)	–0.2	2.6 (–1.4 to 1.2)	6.8 (–2.7 to 4.1)	0.67
	AA	0	0.1 (–0.0 to 0.0)	0.6 (–0.3 to 0.3)	0	0.1 (–0.1 to 0.0)	0.2 (–0.1 to 0.1)	0.353
	EF	0	0.3 (–0.1 to 0.2)	1.6 (–0.6 to 0.9)	0	0.2 (–0.1 to 0.1)	0.7 (–0.4 to 0.3)	0.49
Bergmann2016	IE	–0.1	0.4 (–0.3 to 0.1)	4.1 (–2.2 to 2.0)	–0.1	0.3 (–0.3 to 0.0)	0.9 (–0.6 to 0.2)	0.96
	AA	0	0.3 (–0.1 to 0.2)	1.4 (–0.7 to 0.7)	0.3	0.4 (0.2 to 0.6)	1.2 (–0.3 to 1.0)	< 0.001*
	FE	–0.1	0.8 (–0.5 to 0.3)	4.2 (–2.0 to 2.2)	0.1	0.3 (–0.0 to 0.3)	0.9 (–0.2 to 0.7)	0.038*
TableTop	IE	–0.1	0.5 (–0.4 to 0.1)	4.6 (–2.6 to 2.0)	–0.1	0.4 (–0.3 to 0.0)	0.9 (–0.8 to 0.2)	0.716
	AA	–0.2	3.3 (–2.0 to 1.3)	19.6 (–12.6 to 7.0)	0.2	3.2 (–1.8 to 1.4)	7.5 (–3.7 to 3.7)	0.665
	FE	0.1	0.4 (–0.1 to 0.3)	2.4 (–1.0 to 1.4)	–0.4	0.3 (–0.5 to –0.2)	1.0 (–0.9 to 0.1)	< 0.001*

## Discussion

The A&A method proved to be robust against the spatial alignment of the femur. Significant positional differences were observed for most of the landmarks detected automatically compared to the landmarks identified manually. The differences in the Euclidian distance of the A&A method for the MEC and LEC were comparable to the results of Baek et al.^20^ and Phan et al.^17^. With the exception of the PTC, the differences were considerably larger for landmarks that had been only mapped from the template and were not further refined, such as the SGT and LT. However, the latter two were not used for the construction of the femoral coordinate systems. The PTC showed significant differences for all directions with an exceptionally large difference for the distoproximal direction of 25.6 mm. The cause of these differences is the high positional variability of the PTC and was an imprecise instruction to the observers about the possible location of the PTC. The PTC is defined by the proximal contact point of the femur when placed on a table. Depending on the individual morphology, this point can be located anywhere along the intertrochanteric crest from the lesser to the proximal tip of the greater trochanter. In the instructions to the observers, the PTC was placed exemplary in the middle of the intertrochanteric crest and it was not explicitly pointed out that the most proximal point of the entire intertrochanteric crest was the target. Hence, all observers positioned the PTC on the intertrochanteric crest approximately in the middle between the lesser and posterior greater trochanter. Since the PTC was only used for the calculation of the normal of the TTP, the large mediolateral and distoproximal differences do not affect the TableTop coordinate system. However, the significant difference in the posterioanterior direction causes a significant difference for the flexion–extension of 0.5°. The significant difference of 0.3° for the adduction-abduction for the Bergmann2016 coordinate system resulted from the difference of the neck axis. However, the large dispersion of the neck axes identified manually indicates that the manual detection of the neck isthmus on surface models is unreliable. No significant rotational differences between the manual and A&A method existed for the Wu2002 coordinate system, whereas Kai et al. reported a significant difference between their automatic method and the Wu2002 coordinate system detected manually^29^. The FHC as the origin of the coordinate systems was also affected by significant positional differences of 0.1 mm that should usually be clinically irrelevant. This might also apply for the significant rotational differences of the TableTop and Bergmann2016 coordinate system that did not exceed 0.5°. However, an application-specific evaluation for any use case of the A&A method is recommended. Additional refinements of other landmarks that are biomechanically relevant, such as for the SGT and LT, can be easily integrated into the modular framework. However, the large dispersion of the SGT identified manually indicates that the area of the greater trochanter might be better represented by multiple landmarks.

**Figure 7:**
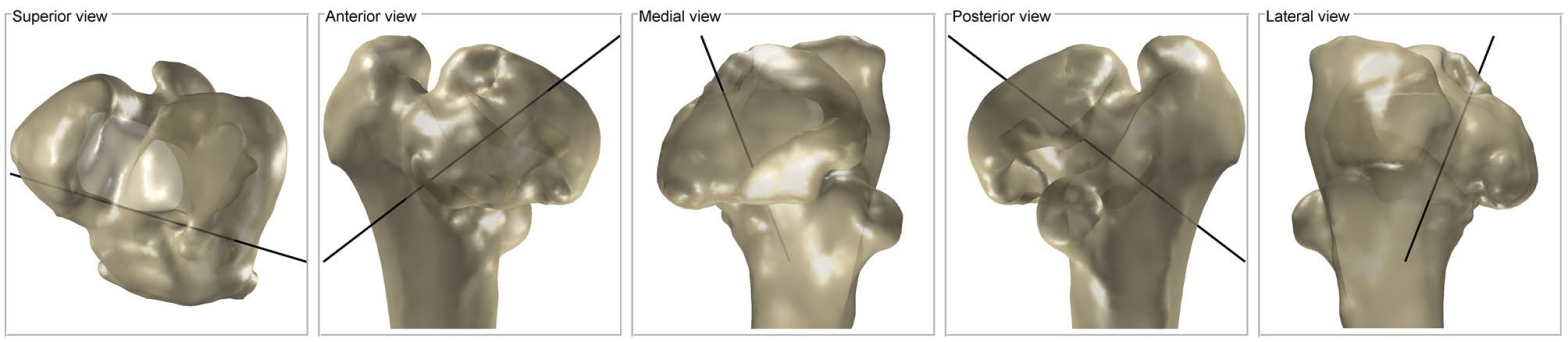
Malalignment of the neck axis (black) detected by the A&A method due to large osteophytes at the femoral neck.

The cadaveric subjects used in this study were free of metal artifacts, very large osteophytes, obvious bone deformities, bone fractures or osteotomies that might cause misdetections of the landmarks. Figure 7 presents a case from a database of total hip arthroplasty patients with a malalignment of the neck axis due to large osteophytes at the femoral neck. A further evaluation of such cases is required.

Some other limitations have to be considered. In addition to the surface model, the side of the femur is required to guarantee that the program is working properly. An incorrect side would probably lead to misdetections or a crash of the program. Furthermore, the surface model has to represent the full femur of the subject due to the registration process of the template. The necessity of specifying the side might be removed and a support for a partial femur might be included in a future version of the program.

## Conclusion

A fully automatic method for the detection of femoral landmarks, axes and planes on surface models for the construction of common bone coordinate systems was developed. To the best of our knowledge, this is first study that comprehensively investigated the difference between the manually and automatically detected femoral coordinate systems. No significant rotational differences existed for the International Society of Biomechanics recommended Wu2002 coordinate system. Significant, though minor rotational differences of maximally 0.5° were present for the TableTop and Bergmann2016 coordinate system. Whether these are acceptable should be evaluated by the potential user regarding the specific application. Future work might include the integration and refinement of additional landmarks and the evaluation of other techniques, such as local region matching^17^ or skeleton extraction^41^.

## Supplementary information

Supplementary Information 1.

Supplementary Information 2.

## Data Availability

All data and code to reproduce the results of this study are openly accessible. A list of the subjects is provided in the Supplementary Table S2. The segmentations and reconstructions are available at https://www.smir.ch. The surface models^47^, the MATLAB code^42–46^ and the manually selected landmarks^47^ are published online. Future updates can be found at https://github.com/RWTHmediTEC/FemoralCoordinateSystem.
