# Supplementary material for: A robust method for automatic identification of femoral landmarks, axes, planes and bone coordinate systems using surface models
Source: Sci Rep. 2020 Nov 30;10:20859. doi: 10.1038/s41598-020-77479-z (PMC7704624; doi:10.1038/s41598-020-77479-z)
Supplement: Supplementary file 1 — Supplementary Information 1. [file 41598_2020_77479_MOESM1_ESM.docx]

[Chair of Medical Engineering](http://www.meditec.rwth-aachen.de/en), Helmholtz-Institute for Biomedical Engineering, RWTH Aachen University, Germany

A robust method for automatic identification of femoral landmarks, axes, planes and bone coordinate systems using surface models

Supplementary Table S1 - List of abbreviations

Maximilian C. M. Fischer, Sonja A. G. A. Grothues, Juliana Habor, Matías de la Fuente, Klaus Radermacher

2020


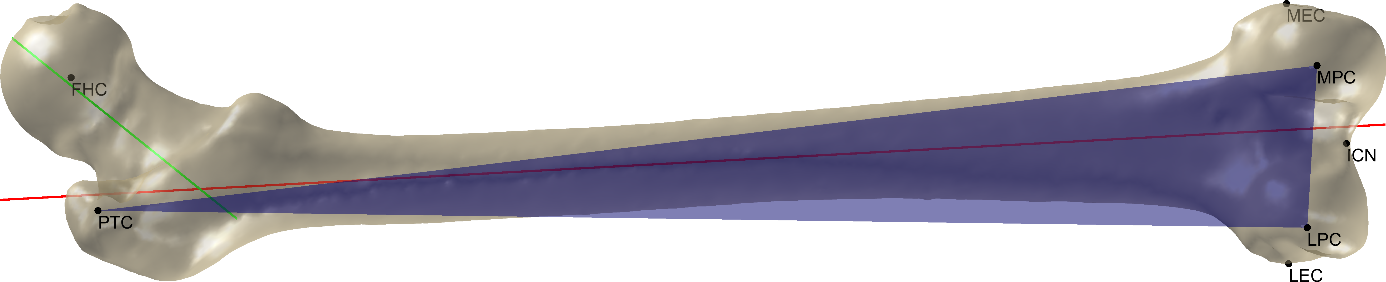


Supplementary Figure 1. The detected landmarks, axes and planes for the construction of the femoral bone coordinate systems. The neck axis is depicted in green, the shaft axis in red and the TTP in blue.

Supplementary Table S1. List of abbreviations.

| **Landmarks, axes, planes** | |
| --- | --- |
| CEA | central elliptic axis |
| EC | epicondyle |
| FHC | femoral head center |
| ICN | intercondylar notch |
| LEC | lateral epicondyle |
| LT | lesser trochanter |
| LPC | lateral posterior condyle |
| MEC | medial epicondyle |
| MPC | medial posterior condyle |
| PFEA | posterior focal elliptic axis |
| PTC | posterior trochanteric crest |
| SGT | superior greater trochanter |
| TTP | table top plane |
| USP | unified sagittal plane |
| **Coordinate systems** | |
| Bergmann2016 | coordinate system used by the OrthoLoad group^1^ |
| TFCS | temporary femoral coordinate system |
| TableTop | coordinate system based on the table top plane^2^ |
| Wu2002 | coordinate system recommended by the International Society of Biomechanics^3^ |
| **Landmark identification methods** | |
| A&A | atlas- and a priori knowledge-based |
| **Error metrics** | |
| AMD | median difference between the landmarks identified by the A&A method and the reference landmarks |
| MMD | median difference between the landmarks identified by the manual method and the reference landmarks |
| **Others** | |
| CT | computed tomography |
| ICP | iterative closest point |
| SICAS | Swiss Institute for Computer Assisted Surgery |

1. Bergmann, G., Bender, A., Dymke, J., Duda, G. & Damm, P. Standardized Loads Acting in Hip Implants. *PloS one* **11,** e0155612; 10.1371/journal.pone.0155612 (2016).

2. Uemura, K., Atkins, P. R. & Anderson, A. E. The effect of using different coordinate systems on in-vivo hip angles can be estimated from computed tomography images. *Journal of biomechanics* **95,** 109318; 10.1016/j.jbiomech.2019.109318 (2019).

3. Wu, G. *et al.* ISB recommendation on definitions of joint coordinate system of various joints for the reporting of human joint motion—part I: ankle, hip, and spine. *Journal of biomechanics* **35,** 543–548; 10.1016/S0021-9290(01)00222-6 (2002).
