# Supplementary material for: A robust method for automatic identification of femoral landmarks, axes, planes and bone coordinate systems using surface models
Source: Sci Rep. 2020 Nov 30;10:20859. doi: 10.1038/s41598-020-77479-z (PMC7704624; doi:10.1038/s41598-020-77479-z)
Supplement: Supplementary file 2 — Supplementary Information 2. [file 41598_2020_77479_MOESM2_ESM.docx]

[Chair of Medical Engineering](http://www.meditec.rwth-aachen.de/en), Helmholtz-Institute for Biomedical Engineering, RWTH Aachen University, Germany

A robust method for automatic identification of femoral landmarks, axes, planes and bone coordinate systems using surface models

Supplementary Table S2 – Information on the cadaveric subjects

Maximilian C. M. Fischer, Sonja A. G. A. Grothues, Juliana Habor, Matías de la Fuente, Klaus Radermacher

2020

**Supplementary Table S2. Used cadaveric subjects from the open source virtual skeleton database (VSDFullBody) hosted at** [**www.smir.ch**](https://www.smir.ch/)**.**

| **Number** | z001 | z009 | z013 | z019 | z023 | z024 | z027 | z035 | z036 | z042 | z046 | z049 | z050 | z055 | z056 | z057 | z061 | z062 | z064 | z066 |
| --- | --- | --- | --- | --- | --- | --- | --- | --- | --- | --- | --- | --- | --- | --- | --- | --- | --- | --- | --- | --- |
| **Age** | 76 | 25 | 41 | 58 | 47 | 57 | 37 | 30 | 62 | 61 | 38 | 34 | 84 | 73 | 26 | 75 | 39 | 43 | 69 | 48 |
| **Gender** | M | M | F | M | F | F | F | F | M | F | M | M | M | M | M | M | F | M | M | M |
